# Case for diagnosis. Dorsal nodule in a 10-year-old male^☆^^☆☆^

**DOI:** 10.1016/j.abd.2020.08.009

**Published:** 2021-01-31

**Authors:** Gongjun Xu, Xuefeng Fu, Jinxian Fang, Chiqing Huang

**Affiliations:** a Department of Dermatology, Jinhua Fifth Hospital, Jinhua, Zhejiang, China; b Department of Dermatology, Jinhua Municipal Central Hospital, Jinhua, Zhejiang, China

**Keywords:** Benign, Cutaneous, Granular cell tumor, S-100 negative

## Abstract

Granular cell tumors (GCTs) are rare soft-tissue neoplasms. GCT immunohistochemistry is positive for S-100, NSE, and CD68. This report describes the case of a 10-year-old male who presented with a dorsal nodule. A biopsy revealed aggregates and sheets of large epithelioid and spindle cells. The cells had abundant eosinophilic granular cytoplasm. Immunohistochemical analysis was positive for CD68, NKI/C3, and synaptophysin; weakly positive for NSE; and negative for S-100, SOX10, HMB45, Melan A, cytokeratin, SMA, EMA, and CD163. The Ki-67 index was less than 1%. A diagnosis of an S-100 negative, cutaneous, benign GCT was determined.

## Case report

**Figure 1:**
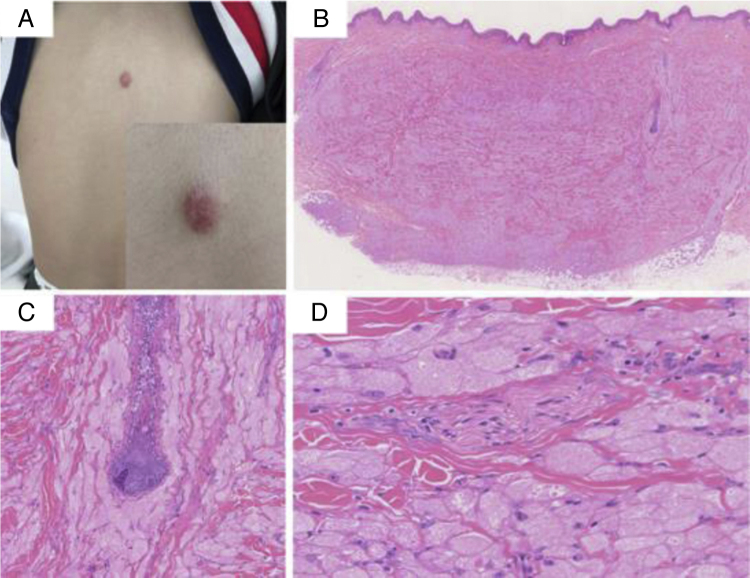
(A) A solitary, round, 3 × 2 cm, dark-red-colored, dome-shaped, firm nodule on the back. (B) At low power, the biopsy showed aggregates and sheets of large cells with distinct cellular borders separated by thin, delicate connective tissue septa. The tumors were covered by a normal epidermis (Hematoxylin & eosin, ×40). (C) The folliculus pili were encased by the tumor (Hematoxylin & eosin, ×100). (D) The cells had abundant eosinophilic granular cytoplasm and occasional deeply eosinophilic round globules. The nuclei were vesicular, with pale chromatin and a single central nucleolus. A nerve fiber tract was also visible (Hematoxylin & eosin, ×200).

**Figure 2:**
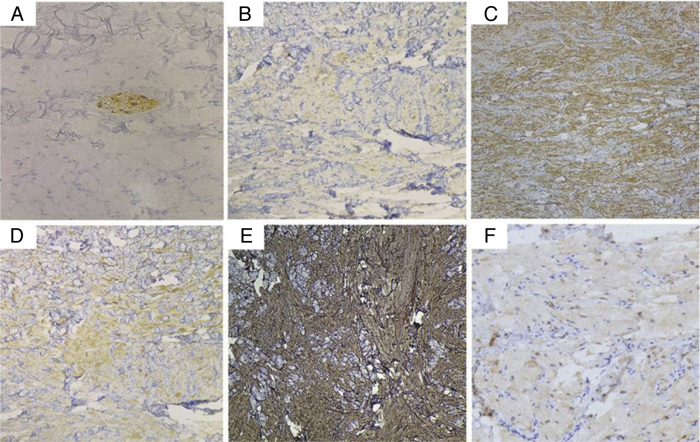
Photomicrographs of immunohistochemical studies. (A) S-100 was negative in the tumor cells, while the nerve fiber tract served as an internal positive control (Original magnification, ×200). (B) CD68, (C) vimentin, (D) Syn, and (E) NKI/C3 (CD63) were positive (Original magnification, ×100), and (F) NSE was weakly positive (Original magnification, ×200).

A 10-year-old male presented with a dorsal nodule, which rapidly increased in size after two months. The lesion was neither painful nor tender. On physical examination, a single, firm, subcutaneous nodular lesion with well-defined borders was noted on the back (Fig. 1a). The subcutaneous nodule was 3 × 2 cm and was adhered to the deep soft tissues. The biopsy revealed aggregates and sheets of large epithelioid and spindle cells, with distinct cellular borders separated by thin, delicate connective tissue septa. The cells had abundant eosinophilic granular cytoplasm and occasional deeply eosinophilic round globules. The nuclei were vesicular with pale chromatin and a single central nucleolus. The skin appendages were encased by the tumor (Fig. 1 b–d). IHC analysis revealed the tumor was positive for CD68, vimentin, and synaptophysin (Syn); weakly positive for NSE; and negative for SOX10, HMB45, Melan A, cytokeratin, SMA, EMA, CD163, CD56, and CD34. In the presence of positive internal and external controls, the tumor cells were negative for S-100 protein. The intracytoplasmic granules were diffuse and strongly positive for NKI/C3 (CD63) (Fig. 2 a–f). The Ki-67 index was less than 1%.

## What is your diagnosis?

a) Tuberous xanthoma
b) Dermatofibroma
c) Leiomyomas
d) S-100-negative cutaneous granular cell tumor

## Discussion

Cutaneous GCTs mostly present as an asymptomatic, slow-growing, solitary nodule, with a multifocal location in 5%–14% of cases.^1^ Histopathologically, GCTs are composed of sheets of large spindle, ovoid, and polygonal cells. At a higher magnification, the cells have abundant, finely granular cytoplasm and small bland nuclei with occasional nucleoli. GCTs show strong and diffuse positivity for the S-100 protein, NSE, CD68, NKI/C3, and vimentin. This staining pattern is suggestive of a Schwann cell origin. However, there is still much debate regarding the histogenesis of GCTs, include striated and smooth muscle cells, perineural fibroblasts, histiocytes, neurogenic cells, undifferentiated mesenchymal cells, and polyphyletic cells.^2^

A distinctive S-100 protein-negative variant of GCT has been observed. In 1991, an originally S-100-negative GCT was reported as a “primitive polypoid granular cell tumor”.^3^ The tumor cells of the present lesion were negative for S-100 protein but stained strongly positive for Syn and weakly for NSE. This staining pattern is suggestive of a possible neuroendocrine origin. CD68 immunoreactivity indicates the presence of lysosomes rather than a marker of histiocytic lineage, as tumor cells are negative for CD163. The intracytoplasmic granules are diffusely and strongly positive for NKI/C3, with a large number of lysosomes in the cytoplasm of the tumor cells.

Some reports have suggested that the absence of S-100 expression may be due to an altered differentiation process in malignant GCTs.^4^ Although the present case did not present malignant features clinically or histopathologically, an appropriate follow-up should be conducted to allow further characterization of the behavior of this rare neoplasm.

## Financial support

None declared.

## Authors’contributions

Gongjun Xu: Conception and planning of the study; elaboration and writing of the manuscript; critical review of the manuscript.

Xuefeng Fu: Conception and planning of the study; elaboration and writing of the manuscript.

Jinxian Fang: Design and planning of the study; preparation and writing of the manuscript.

Chiqing Huang: Effective participation in research orientation; critical review of the literature.

## Conflicts of interest

None declared.
